# Predictors for non-response to PROMs across three areas of inpatient rehabilitation – a nationwide cross-sectional study in Switzerland

**DOI:** 10.1186/s41687-026-01183-1

**Published:** 2026-08-25

**Authors:** Frederike Basedow, Gavin Brupbacher, Anke Scheel-Sailer, Thomas Sigrist, Jan Vontobel, Manuela Marquardt, Marie Utsch, Gaia Garuffi, Stephan Tobler, Anika Zembic

**Affiliations:** 1 Institute of Medical Sociology and Rehabilitation Science, Charité - University Medical Center, Corporate Member of Freie Universität Berlin and Humboldt-Universität zu Berlin, Berlin, Germany; 2 OBERWAID AG, St. Gallen, Switzerland; 3 Centre for Rehabilitation and Sport Medicine, Inselspital, University Hospital of Bern, Bern, Switzerland; 4 Berner Reha Zentrum, Bern, Switzerland; 5 Department for Pulmonary Medicine, Klinik Barmelweid, Barmelweid, Switzerland; 6 Hochgebirgsklinik Davos, Davos, Switzerland; 7 ANQ, Bern, Switzerland

**Keywords:** Non-response, Patient-reported outcome measures, Rehabilitation, Health equity, Quality assurance, MacNew Heart, Chronic Respiratory Disease Questionnaire (CRQ), Patient-Health Questionnaire 15 (PHQ-15), Hospital Anxiety and Depression Scale (HADS)

## Abstract

**Background:**

Patient-reported outcome measures (PROMs) are valuable tools to integrate the patient perspective and improve quality of rehabilitation services. However, there is a risk of non-response bias. Identifying characteristics associated with non-response is important to reduce barriers to PROM completion and to ensure quality care for all patients undergoing rehabilitation.

**Methodology:**

In this cross-sectional observational study, we used routine data from the Swiss national quality assurance program for inpatient rehabilitation, in which PROMs are employed at admission and discharge for all patients in cardiac, psychosomatic and pulmonary rehabilitation. We included all patients aged 18 years or older, who completed cardiac, pulmonary or psychosomatic rehabilitation between 01.01.2016 and 31.12.2022. We used mixed-effects logistic regression to identify predictors for non-response to PROMs across the three areas of rehabilitation. Non-response was defined as recorded refusal to complete the PROMs (cardiac: MacNew Heart, psychosomatic: Hospital Anxiety and Depression Scale (HADS) and Patient-Health Questionnaire (PHQ)-15, pulmonary: Chronic Respiratory Disease Questionnaire CRQ) at admission and/or discharge. Several socio-demographic and medical characteristics, as well as clinic and year were included as covariates in the regression model.

**Results:**

We included 39,630 patients completing the MacNew Heart, 12751 HADS, 13195 PHQ and 16193 completing the CRQ. Non-response rates were highest for the MacNew Heart (28.6%) and CRQ (25.0%) and lower for the HADS (14.4%) and PHQ (17.5%). Across all three PROMs we observed strong significant associations of a nationality-based proxy for being a non-native speaker, admission from or discharge into a nursing home/rehabilitation and discharge into acute care after rehabilitation with non-response. Having no supplementary health insurance increased the odds for non-response on the MacNew Heart, HADS and CRQ. Additionally, higher comorbidity Cumulative Illness Rating Scale (CIRS) score and age were also significantly associated with non-response. Between-clinic differences explained a substantial proportion of variance in non-response, with adjusted ICCs ranging from 0.34 in psychosomatic to 0.62 in cardiac rehabilitation.

**Conclusion and relevance:**

Non-response to PROMs in rehabilitation is predicted by impaired health, older age, and a proxy for non-native language skills. This should be addressed by reducing barriers for patients with these characteristics to reduce bias and to ensure health equity.

**Supplementary Information:**

The online version contains supplementary material available at https://doi.org/10.1186/s41687-026-01183-1.

## Background

Patient-reported outcome measures (PROMs) are standardized, validated questionnaires assessing the self-reported health status of patients. Among others, they measure patients’ perspective on physical functioning, emotional and psychosocial well-being, or overall health-related quality of life (HRQOL) [27]. PROMs have been widely recognized as valuable tools to integrate patients’ perspective and improve quality in clinical care. They facilitate shared decision-making, enhance early recognition and monitoring of symptoms, enable individualized treatment adjustments, and improve satisfaction and communication between patients and healthcare professionals [8, 17, 20, 28, 32, 35]. PROMs are also increasingly employed in quality assurance programs to monitor the quality of health care delivery, benchmark treatment outcomes, and inform operations and policies [6, 11, 19, 29]. However, since PROMs rely on self-assessments, they are subject to non-response and therefore missing data. This can lead to a bias if certain groups of patients are systematically underrepresented.

In Switzerland, PROMs are used in the nationwide quality evaluation of cardiac, pulmonary and psychosomatic rehabilitation clinics. The MacNew Heart questionnaire [14, 23, 36] and the Chronic Respiratory Disease Questionnaire (CRQ) [13] are used to assess HRQOL in cardiac and pulmonary rehabilitation, respectively. In psychosomatic rehabilitation, the somatic symptoms module of the Patient-Health Questionnaire (PHQ-15) [21] and the Hospital Anxiety and Depression Scale (HADS) [39] were used until 2023 as measures of somatic and mental health^1^. Non-response rates between 12% and 29% have been observed for these PROMs in the yearly routine evaluations across Switzerland, with lower rates for PHQ-15 and HADS and higher rates for CRQ and MacNew Heart [3–5]. Identifying factors that contribute to non-response for these instruments is important to ensure valid results and to prevent bias in outcome evaluations and quality assurance comparisons across clinics.

### Objective

We recently identified the following predictors for non-response in cardiac rehabilitation in Switzerland [19]: Female sex, higher age, non-native language background, higher disease burden, and not having supplementary health insurance.

The aim of this study was to extend these findings, including data from more recent years as well as to identify and compare predictors for non-response to PROMs in psychosomatic and pulmonary rehabilitation.

## Methods

The reporting of our study follows the Strengthening the Reporting of Observational Studies in Epidemiology (STROBE) checklist [38].

### Study design and setting

This cross-sectional study used data from the Swiss national quality assurance program for inpatient rehabilitation implemented in 2013 by ANQ, the competence center for quality measurements in Swiss hospitals and clinics. ANQ collects data for all inpatient rehabilitation stays in Switzerland. Depending on the type of rehabilitation, different instruments are used to assess physical functioning and HRQOL. All instruments are administered directly to the patients within the first three days after admission and at discharge. The ANQ provides language versions of all PROMs in German, French and Italian. The process of PROM administration lies within the clinic’s responsibility, and no further requirements are specified by ANQ. Additionally, socio-demographic and basic medical information are collected by clinical staff at admission, including comorbidities using the cumulative illness rating scale (CIRS) [24, 26, 33]. More details on the rehabilitation quality assurance program and data collection can be found on the ANQ website [2]. Data was available for cardiac and pulmonary rehabilitation from 2016 to 2022, and for psychosomatic rehabilitation from 2017 to 2022. The data is recorded at the level of individual rehabilitation stays rather than individual patients.

### Participants

We included all cases of patients 18 years or older, who completed their inpatient rehabilitation stay in a clinic in Switzerland in cardiac, psychosomatic or pulmonary rehabilitation. Rehabilitation stays that were shorter than 7 days or discontinued (e.g. due to death or transfer into acute care) were excluded. Additionally, only patients who were admitted from and discharged to another care facility (acute clinic, nursing home or rehabilitation clinic) or home were included, and all socio-demographic and basic clinical information had to be complete.

### Variables

The outcome variable in this study was non-response to at least one assessment of the MacNew Heart questionnaire in cardiac rehabilitation, to the PHQ-15 and HADS in psychosomatic rehabilitation, and to the CRQ in pulmonary rehabilitation (see Supplement 1). Patients’ refusal or inability to complete the PROMs, including the reason, is documented by clinical staff.

Patients were categorized as non-responders, if non-response was recorded either at admission and/or discharge. Along with documented non-response, all items were required to be left empty.

Responders were patients without a documented non-response and with complete measurements at both time points. Complete measurements were cases who had filled out enough items so that a score could be calculated (see Supplement 1). Patients without a recorded non-response along with missing or incomplete measurements were excluded from the study as data validity for these patients cannot be guaranteed. Accordingly, the term “non-response” in this study refers to documented refusal or inability to complete the PROM questionnaire and does not include patients with missing or inconsistently documented PROM records.

The following socio-demographic variables were used to describe the patient population and were included as covariates in the regression models: age (years), sex (female/male), supplementary health insurance (yes/no), and a proxy for Swiss language proficiency (French, Italian or German as first language (L1) or not). Supplementary health insurance includes any kind of additional insurance benefits that patients may purchase on top of statutory health insurance. Information on language proficiency was not available and therefore approximated from the nationality of patients. In Switzerland, French, Italian and German are the three main official languages (along with Romansh, which was not regarded in this study). Patients from any country with at least one of these languages as official language were categorized as L1 speakers, while citizens from countries without any of these languages as official language were categorized as non-native language speakers.

Additionally, we included the main discharge diagnosis (grouped into disease categories, see Supplementary Tables 1–3), length of rehabilitation stay, location before admission and after discharge (both categorized into home, nursing home/rehabilitation, acute care), and total CIRS score (in points 0–56, higher scores indicate higher comorbidity) to capture the health status of patients.

### Statistical methods

Statistical analyses and graphical representations were done in R version 4.2.2. using *data.table*, *lme4*, *performance*,* ggeffects*, and *ggplot2* packages.

For each PROM-subsample, we stratified the number of patients into non-responders and responders and described the distribution (mean and standard deviations (SD)) of demographic and clinical patient characteristics.

A mixed-effects logistic regression model was run for each PROM sample with non-response as the binary outcome variable. Clinic was used as a random-effects variable in all models. All demographic and clinical characteristics as described above were included as covariates in the analysis. Year was included to control for variance attributable to period effects – for example due to the COVID-19 pandemic. Age, length of the rehabilitation stay, and CIRS score were entered as continuous variables. Binned residual plots from the *performance* package were used to determine the model fit for the whole model, as well as stratified by each continuous variable. If less than 80% of the residuals were inside error bounds, the model fit was considered insufficient and B-splines with 5 knots were applied to each variable displaying a bad fit. This was the case for the length of rehabilitation stay in all 4 models as well as for age in the MacNew Heart model.

Odds ratios (OR), corresponding 95%-confidence intervals (CI) and p-values were calculated for each variable. Additionally, we calculated the odds and corresponding CI for non-response from the predicted probability using *ggeffects* for each patient and plotted it against the distribution of each continuous variable in order to graphically analyze the association of age, length of rehabilitation stay and CIRS score with non-response. The Intraclass correlation coefficient (ICC) was calculated for each model to assess the proportion of variance explained at the clinic-level.

## Results

### Participants

**Fig. 1:**
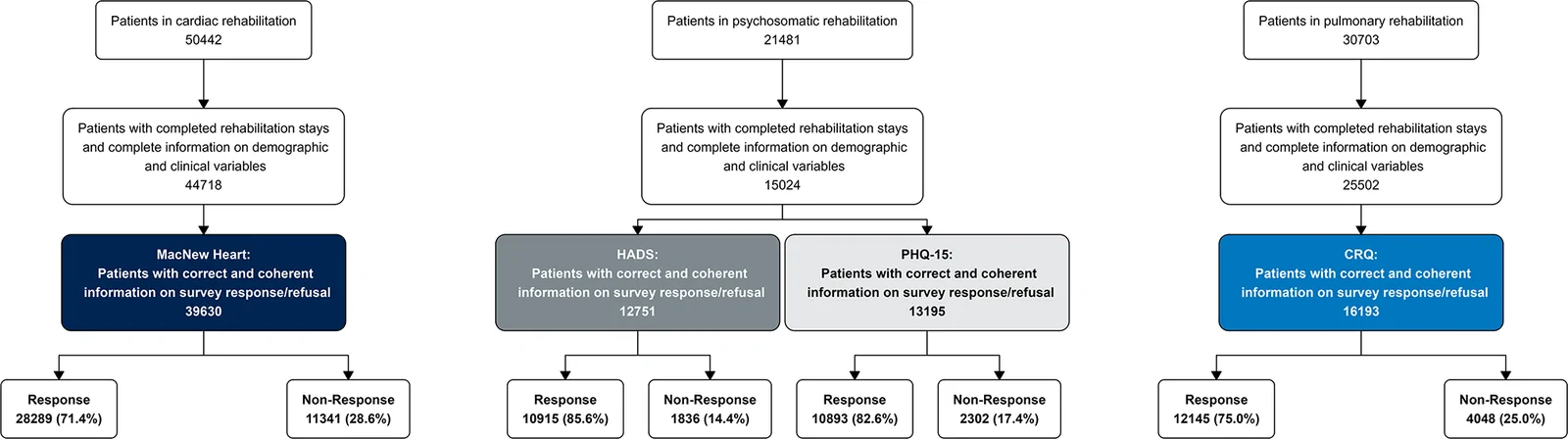
Flowchart for patient selection

The dataset contained a total of 50442 patients in cardiac rehabilitation, 30703 in pulmonary rehabilitation, and 21481 in psychosomatic rehabilitation. After the application of inclusion and exclusion criteria, the number of patients included for further analyses was 39630 for MacNew Heart, 12,751 for HADS, 13195 for PHQ-15 and 16193 for CRQ (Fig. 1).

In total, data from 34 clinics was included in the analysis (22 clinics with cardiac, 15 with psychosomatic, and 16 with pulmonary rehabilitation).

### Descriptive data

The patient population differed in terms of demographic and clinical characteristics between the three rehabilitation domains. In cardiac and pulmonary rehabilitation there was a higher proportion of male than female patients (MacNew Heart: 70% male; CRQ: 55% male), whereas in psychosomatic rehabilitation, the majority of patients was female (HADS and PHQ: 65% female). Furthermore, most patients in cardiac and pulmonary rehabilitation came directly from acute care into the clinic (MacNew Heart: 93%; CRQ: 82%) while psychosomatic patients were predominantly at home before their rehabilitation stays (12% acute care). Additionally, psychosomatic patients had on average lower CIRS comorbidity scores (HADS: mean = 9.15, SD = 5.36; PHQ: mean = 9.15, SD = 5.33; MacNew Heart: mean = 17.07, SD = 6.45; CRQ: mean = 15.49, SD = 6.65) and rehabilitation stays that were approximately 10 days longer than for cardiac and pulmonary patients (HADS: mean = 31.42, SD = 16.72; PHQ: mean = 31.24, SD = 16.60; MacNew Heart: mean = 21.57, SD = 5.77; CRQ: mean = 21.73, SD = 7.32) (Table 1). Socio-demographic and clinical characteristics of excluded patients that could not be reliably assigned to response and non-response groups can be found in Supplementary Table 7.

**Table 1:** Descriptive characteristics by response status and instrument/PROM

	Cardiology: Mac New Heart			Psychosomatics: HADS			Psychosomatics: PHQ			Pulmonology: CRQ		
	Total	non-resp.	resp.	Total	non-resp.	resp.	Total	non-resp.	resp.	Total	non-resp.	resp.
***n*** ** (%)**	39630	11341 (28.62)	28289 (71.38)	12751	1836 (14.40)	10915 (85.60)	13195	2302 (17.45)	10893 (82.55)	16193	4048 (25.00)	12145 (75.00)
**Female Sex ** ***n*** ** (%)**	12039 (30.38)	3769 (33.23)	8270 (29.23)	8267 (64.83)	1224 (66.67)	7043 (64.53)	8532 (64.66)	1504 (65.33)	7028 (64.52)	7305 (45.11)	1795 (44.34)	5510 (45.37)
**Mean Age (SD)**	68.29 (11.72)	69.36 (12.03)	67.86 (11.57)	50.39 (13.13)	51.80 (13.87)	50.16 (12.99)	50.36 (13.11)	51.50 (13.86)	50.11 (12.94)	68.11 (10.96)	68.63 (11.35)	67.94 (10.82)
**L1 Speaker ** ***n*** ** (%)**	37938 (95.73)	10359 (91.34)	27579 (97.49)	11452 (89.81)	1524 (83.01)	9928 (90.96)	11792 (89.37)	1893 (82.23)	9899 (90.87)	15130 (93.44)	3452 (85.28)	11678 (96.15)
**Pre-rehabilitation location: Home ** ***n*** ** (%)**	2360 (5.96)	742 (6.54)	1618 (5.72)	11104 (87.08)	1494 (81.37)	9610 (88.04)	11431 (86.63)	1829 (79.45)	9602 (88.15)	2864 (17.69)	593 (14.65)	2271 (18.70)
**Pre-rehabilitation location: Nursing home/ Rehabilitation ** ***n*** ** (%)**	499 (1.26)	160 (1.41)	339 (1.20)	149 (1.17)	33 (1.80)	116 (1.06)	165 (1.25)	51 (2.22)	114 (1.05)	84 (0.52)	35 (0.86)	49 (0.40)
**Pre-rehabilitation location: Acute care ** ***n*** ** (%)**	36771 (92.79)	10439 (92.05)	26332 (93.08)	1498 (11.75)	309 (16.83)	1189 (10.89)	1599 (12.12)	422 (18.33)	1177 (10.81)	13245 (81.79)	3420 (84.49)	9825 (80.90)
**Post-rehabilitation location: Home ** ***n*** ** (%)**	38839 (98.00)	10903 (96.14)	27936 (98.75)	12542 (98.36)	1740 (94.77)	10802 (98.96)	12967 (98.27)	2183 (94.83)	10784 (99.00)	15558 (96.08)	3753 (92.71)	11805 (97.20)
**Post-rehabilitation location: Nursing home/ Rehabilitation ** ***n*** ** (%)**	374 (0.94)	194 (1.71)	180 (0.64)	88 (0.69)	26 (1.42)	62 (0.57)	96 (0.73)	36 (1.56)	60 (0.55)	394 (2.43)	174 (4.30)	220 (1.81)
**Post-rehabilitation location: Acute care ** ***n*** ** (%)**	417 (1.05)	244 (2.15)	173 (0.61)	121 (0.95)	70 (3.81)	51 (0.47)	132 (1.00)	83 (3.61)	49 (0.45)	241 (1.49)	121 (2.99)	120 (0.99)
**Supplementary or private health insurance n (%)**	10904 (27.51)	2368 (20.88)	8536 (30.17)	1358 (10.65)	169 (9.20)	1189 (10.89)	1389 (10.53)	210 (9.12)	1179 (10.82)	3196 (19.74)	586 (14.48)	2610 (21.49)
**Mean length of stay in days (SD)**	21.57 (5.77)	21.92 (6.46)	21.42 (5.47)	31.42 (16.72)	31.44 (19.34)	31.42 (16.24)	31.24 (16.60)	30.33 (18.13)	31.43 (16.25)	21.73 (7.32)	21.07 (7.71)	21.95 (7.17)
**Mean CIRS Score (SD)**	17.07 (6.45)	18.05 (6.34)	16.68 (6.45)	9.15 (5.36)	10.24 (6.06)	8.97 (5.21)	9.15 (5.33)	9.97 (5.63)	8.98 (5.25)	15.49 (6.65)	16.07 (6.67)	15.30 (6.63)

### Outcome data

The non-response rate was highest for the MacNew Heart with 28.6% missing measurements, followed by the CRQ with 25.0%. Lower rates were observed in psychosomatic rehabilitation, with 14.4% non-response for the HADS and 17.5% for the PHQ-15 (Table 1; Fig. 1). The number of cases with non-response at admission, discharge or both for each PROM can be found in Supplementary Table 4.

In all four samples, non-responders were older and less often native speakers of German, French or Italian than responders. They were more often discharged into acute care after rehabilitation and had a higher score on the CIRS-comorbidity scale than the response groups. Furthermore, the proportion of patients with supplementary health insurance was lower in the non-response than in the response groups (Table 1, Supplementary Table 5).

### Main results

**Fig. 2:**
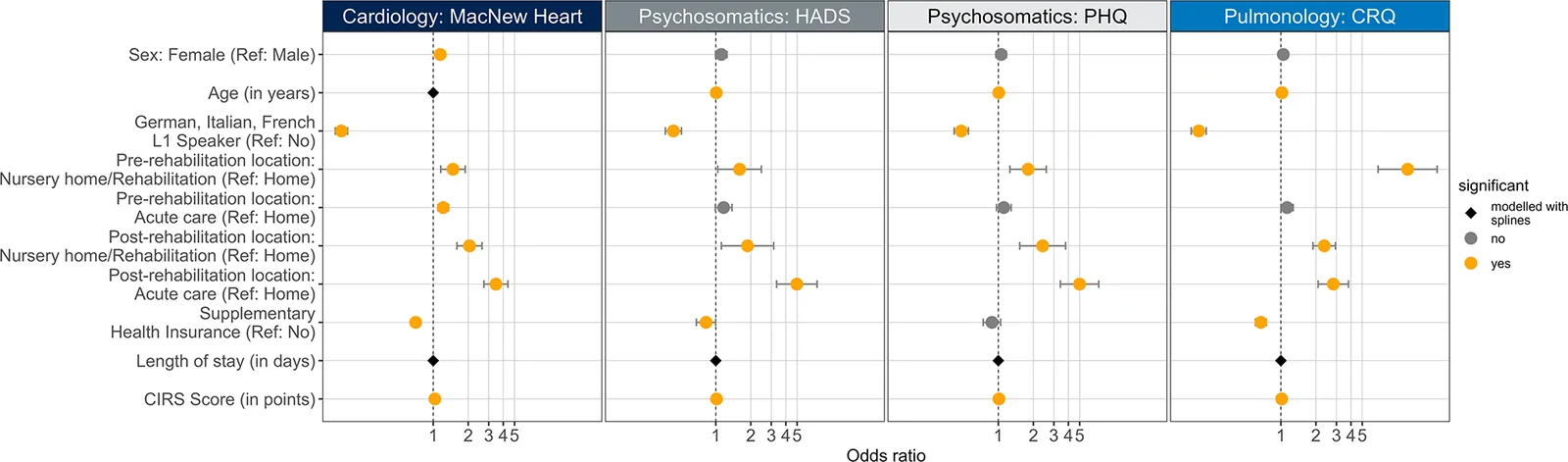
Odds ratios for non-response to MacNew Heart, HADS, PHQ-15 and CRQ by covariates

The regression models for all four instruments revealed similar predictors for non-response across the four different PROMs. Having a non-native language background (inverted OR (95%-CI)): MacNew Heart: 6.3 (5.6–7.1); HADS: 2.3 (2.0-2.7); PHQ: 2.1 (1.8–2.4); CRQ: 5.0 (4.4–5.9); see Table 2 for original ORs) as well as being discharged into a nursing home/rehabilitation (OR (95% CI): MacNew Heart: 2.1 (1.6–2.6); HADS: 1.9 (1.1–3.1); PHQ: 2.4 (1.5–3.8); CRQ: 2.4 (1.9–2.9)) or acute care (OR (95% CI): MacNew Heart: 3.5 (2.7–4.4); HADS: 5.0 (3.3–7.4); PHQ: 5.0 (3.4–7.3); CRQ: 2.8 (2.1–3.8)) after rehabilitation showed strong associations with non-response. Furthermore, being admitted from a nursing home/rehabilitation was associated with non-response to all PROMs (OR (95% CI): MacNew Heart: 1.5 (1.2–1.9); HADS: 1.6 (1.0-2.5); PHQ: 1.8 (1.3–2.6); CRQ: 12.3 (6.8–22.0)), while admission from acute care showed significantly higher odds for non-response to the MacNew Heart but not the other PROMs (OR (95% CI): MacNew Heart: 1.22 (1.1–1.35); HADS: 1.17 (0.99–1.38); PHQ: 1.11 (0.96–1.29); CRQ: 1.13 (1–1.28)). The comparatively large odds ratio observed for the CRQ should be interpreted with caution, as it was based on a relatively small subgroup of patients admitted from a nursing home/rehabilitation facility. Having no supplementary health insurance was also associated with higher odds for non-response to the MacNew Heart, HADS, and CRQ, but not the PHQ (inverted OR (95% CI): MacNew Heart: 1.4 (1.3–1.5); HADS: 1.2 (1.0-1.5); PHQ: 1.1 (1.0-1.4); CRQ: 1.5 (1.3–1.7); see Table 2 for original ORs). Furthermore, each additional point on the CIRS (OR (95% CI): MacNew Heart: 1.03 (1.03–1.04); HADS: 1.02 (1.01–1.03); PHQ: 1.01 (1.00-1.02); CRQ: 1.02 (1.01–1.02)) and each year of age (OR (95% CI): HADS: 1.01 (1.01–1.01); PHQ: 1.01 (1.00-1.01); CRQ: 1.02 (1.02–1.02)) also significantly increased the risk of non-response (Fig. 2; Table 2).

**Fig. 3:**
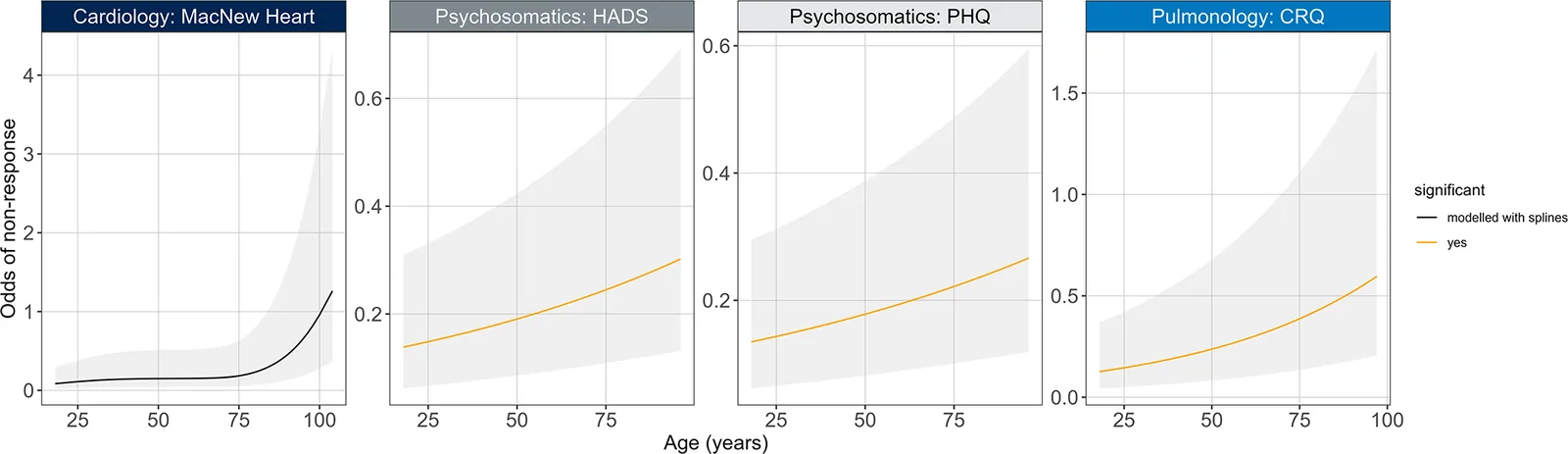
Predicted odds for non-response to MacNew Heart, HADS, PHQ-15 and CRQ by age

Graphical analysis of the continuous covariates confirmed an increase in the odds for non-response with higher age and CIRS-score. For age, the association was approximately linear for the HADS, PHQ and CRQ, while a more pronounced increase in the odds of non-response at higher ages was observed for the MacNew Heart. (Fig. 3, Supplementary Fig. 1).

For the MacNew Heart, we observed significantly higher probabilities for non-response in women compared to men. No significant association was seen between sex and non-response for PROMs in psychosomatic and pulmonary rehabilitation. For all four instruments, there was no clear link between the length of rehabilitation stay and non-response (Fig. 2, Supplementary Fig. 2).

Some disease categories were significantly associated with non-response. For the MacNew Heart, we saw lower odds for non-response in patients with valve disorders, while odds were higher in patients with other unspecified diseases of the heart, the arteries or the circulatory system (Ref: Chronic Ischemic Heart Disease). For both HADS and PHQ in psychosomatic rehabilitation, odds for non-response were higher in patients with severe depressive episodes, musculoskeletal disorders, and other unspecified mental and somatic illnesses (Ref: Addiction disorders). Headache was also strongly associated with non-response on the HADS but not the PHQ. For the CRQ, we saw lower odds for non-response in patients with COPD, oncological diseases and other unspecified pneumological diseases (Ref: Flu & Pneumonia) (Supplementary Table 6). For all four PROMs, there were some years with significantly higher or lower rates of non-response compared to the first year included in the analysis. For the MacNew Heart the odds for non-response were higher in 2020 while in all other years the odds for non-response were lower or similar to 2016. For the HADS we saw higher odds for non-response in 2021 and lower odds in 2018 and 2019 compared to 2017. For the PHQ, the years 2018, 2020 and 2021 were associated with a higher risk for non-response compared to 2017. For the CRQ, the odds for non-response were lower in 2017 to 2019 compared to 2016 but higher in 2021 and 2022 (Supplementary Table 6).

The adjusted ICC was 0.34 in the models for the PROMs in psychosomatic rehabilitation, 0.54 in the CRQ model and 0.62 in the MacNew Heart model. This indicates that one third of the variance in the odds for non-response in psychosomatic rehabilitation and more than half of the variance in cardiac and pulmonary rehabilitation can be explained by differences at the clinic level. The R² was 0.64, 0.41, 0.395, and 0.58 for the models of the MacNew Heart, HADS, PHQ and CRQ, respectively.

**Table 2:** Odds ratios (OR) and confidence intervals (CI) for non-response by instrument/PROM

	Cardiology: Mac New Heart		Psychosomatics: HADS		Psychosomatics: PHQ		Pulmonology: CRQ	
	OR (2.5% - 97.5% CI)	p-value	OR (2.5% - 97.5% CI)	p-value	OR (2.5% - 97.5% CI)	p-value	OR (2.5% - 97.5% CI)	p-value
Intercept	0.84 (0.24 - 2.88)	0.777	0.92 (0.40 - 2.1)	0.839	0.85 (0.38 - 1.90)	0.694	0.73 (0.25 - 2.15)	0.568
Female Sex (Ref.: Male)	1.15 (1.09 - 1.21)	<0.001	1.12 (0.99 - 1.25)	0.062	1.06 (0.96 - 1.17)	0.279	1.05 (0.97 - 1.13)	0.249
Age (not displayed if modelled with splines)	-	-	1.01 (1.01 - 1.01)	<0.001	1.01 (1.00 - 1.01)	<0.001	1.02 (1.02 - 1.02)	<0.001
L1 Speaker (Ref.: no L1 speaker)	0.16 (0.14 - 0.18)	<0.001	0.43 (0.37 - 0.51)	<0.001	0.48 (0.42 - 0.55)	<0.001	0.20 (0.17 - 0.23)	<0.001
Pre-rehabilitation location: Nursery Home/ Rehabilitation (Ref.: Home)	1.48 (1.16 - 1.88)	0.002	1.60 (1.04 - 2.47)	0.032	1.80 (1.26 - 2.58)	0.001	12.28 (6.84 - 22.02)	<0.001
Pre-rehabilitation location: Acute care (Ref.: Home)	1.22 (1.10 - 1.35)	<0.001	1.17 (0.99 - 1.38)	0.068	1.11 (0.96 - 1.29)	0.146	1.13 (1.00 - 1.28)	0.051
Post-rehabilitation location: Nursery Home/ Rehabilitation (Ref.: Home)	2.05 (1.60 - 2.63)	<0.001	1.87 (1.12 - 3.14)	0.017	2.40 (1.53 - 3.79)	<0.001	2.36 (1.89 - 2.94)	<0.001
Post-rehabilitation location: Acute care (Ref.: Home)	3.46 (2.73 - 4.39)	<0.001	4.97 (3.33 - 7.42)	<0.001	4.99 (3.41 - 7.29)	<0.001	2.82 (2.09 - 3.81)	<0.001
Supplementary or private health insurance (Ref.: statutory)	0.71 (0.67 - 0.75)	<0.001	0.82 (0.68 - 1.00)	0.047	0.88 (0.74 - 1.05)	0.148	0.67 (0.60 - 0.75)	<0.001
Length of stay in days (not displayed if modelled with splines)	-	-	-	-	-	-	-	-
CIRS Score	1.03 (1.03 - 1.04)	<0.001	1.02 (1.01 - 1.03)	0.003	1.01 (1.00 - 1.02)	0.013	1.02 (1.01 - 1.02)	<0.001

## Discussion

### Key results

The aim of this study was to compare predictors for non-response across four different PROMs in cardiac, psychosomatic, and pulmonary inpatient rehabilitation. Our results show that older age, a proxy for non-native language skills, being admitted from or discharged into a nursing home or rehabilitation clinic, discharge into acute care and a higher CIRS score are robust predictors of non-response for all PROMs used in Swiss inpatient rehabilitation. The largest effects were found for non-native language background and discharge into acute care, which was included as a proxy for poor health. Not having supplementary health insurance was also associated with non-response to three of the four PROMS. Supplementary health insurance is purchased in addition to statutory health insurance and may be associated with greater financial resources. Thus, the observed association may indicate that patients with potentially higher disease burden and lower financial resources are more likely to not complete PROMs. To reduce the risk of non-response bias, barriers should be lowered for patients with these characteristics when administering PROMs. Moreover, our findings indicate that a substantial portion of non-response to PROMs is related to clinic-level factors, highlighting the importance of provider- or clinic-level influences on PROM completion.

### Generalizability

Our findings replicate the results of our previous study, in which higher age, non-native language background, not having supplementary health insurance, discharge into acute care and higher CIRS scores were also associated with non-response for the MacNew Heart questionnaire. A higher likelihood of non-response with older age, limited language skills, lower socioeconomic status and higher disease burden has also been shown in the literature [7, 10, 16, 22, 25, 34, 37].

Additionally, non-response for the MacNew Heart questionnaire was linked to female sex, which also confirmed the results of our previous study. However, this finding was not apparent for PROMs in other areas of rehabilitation. The association between sex and non-response is unclear from previous literature. While some research in acute care also found that women were less likely to fill out PROMs [37], others have found a higher chance of non-response for male patients [1, 18, 22]. Other studies did not find a link between sex and non-response [7, 10, 34].

In contrast to our previous study, in which longer rehabilitation stays predicted non-response, we did not find such an association in the current study. Instead of strict parametric modelling and interpreting p-values, we used B-splines to model rehabilitation length and assessed its relation to non-response graphically, which limits direct comparability to the previous results. We chose this alternative method to ensure proper fitting of the current data. Nevertheless, the results are surprising, as the length of rehabilitation may serve as a proxy for health status (more impaired patients stay longer in rehabilitation) [9] and an association between higher disease burden and non-response is well established [7, 18, 19, 22, 25]. However, the length of stay is often predetermined in rehabilitation. Other factors that represent health status, such as comorbidities or functioning tests, are more relevant to consider when assessing who may need extra support with filling out PROMs.

In addition to patient characteristics, the facility in which patients are treated was associated with PROM completion. This was indicated by varying non-response rates across all clinics as well as by the high ICC in all models. This is in line with our previous study [19]. As we do not have information on clinical conditions, such as the ratio of staff to patients, we were not able to include this in our analysis to identify facility characteristics that are associated with non-response. However, previous research has shown that limited time of health-care staff, time constraints for patients to fill out questionnaires, inadequate IT-infrastructure, or administering PROMs on tablets [25, 27] are barriers to PROM-completion. Whether these and other facility characteristics play a role in non-response on PROMs in Swiss rehabilitation is an interesting topic for future research.

It is important to note, that our study defined non-response as refusal or inability to complete the PROM assessment at either admission or discharge. This definition was chosen because, in the context of quality assurance, both measurements are required to evaluate changes in patient-reported outcomes. Consequently, the non-response group also included individuals who completed the PROM at one of the two time points. The identified predictors should therefore be interpreted within the context of quality assurance and may not be directly generalizable to settings in which only a single PROM assessment is required.

### Strengths and limitations

The data used in this study bears some limitations. Our selection of covariates was limited to variables that are routinely collected and delivered as part of the ANQ quality assurance program. Hence, we were not able to include other potentially relevant factors, such as education or more detailed information on socio-economic status, in our analysis. We also did not have information on actual language skills of the patients and only used a proxy based on the patients’ citizenship, which may not fully represent actual fluency in Italian, French or German. This approach may have introduced a bias by including individuals with fluent language skills despite their nationality, potentially underestimating the true effect. Furthermore, we were not able to control for clinic-level differences in PROM administration, such as staff availability, IT-infrastructure, documentation quality, whether PROMs were administered on paper or tablet and whether different language versions were offered. As discussed above, these factors may also have contributed to the observed non-response rates. Additionally, we cannot identify individual patients in the data and it is possible that some patients were included with multiple rehabilitation stays. This may have overestimated our results.

There are also some limitations to our analytical approach. We excluded all cases with incomplete PROM measurements, as well as cases for which a non-response was documented alongside item responses, because we could not ensure data validity for the cases and reliably assign them to either group. The excluded cases differed from the included cases with regard to several socio-demographic and clinical characteristics, indicating that the study sample may not be fully representative of the underlying rehabilitation population. In particular, a large number of CRQ cases was excluded due to ambiguous response documentation, which may have introduced a selection bias and may limit the generalizability of the findings. Furthermore, we used the post-rehabilitation location and length of stay as proxies for health status in our model, assuming that they reflect the patient’s overall condition. However, these variables are not known before the rehabilitation stay and their association with non-response, which was partly assessed at admission, should be interpreted with caution. Finally, the observational cross-sectional design of our study precludes causal inference. Hence, the identified predictors should be interpreted as associations rather than causal determinants of non-response.

Despite these limitations, the data set is a valuable source for the study of non-response, as it contains demographic and clinical information for all patients, irrespective of PROM-completion. It furthermore consists of information on where patients were treated, which made it possible to distinguish between individual-level and facility-level influences on non-response. Moreover, since the data comprises information for all patients undergoing inpatient rehabilitation in Switzerland, it allows us to study non-response on national level. Finally, the well-established and long-term use of PROMs in three rehabilitation domains in Switzerland enabled us to identify robust predictors of non-response across different fields and time periods that are likely generalizable to PROMs in other areas of rehabilitation.

### Interpretation

Our findings show that patients with more impaired health and older age have a higher chance of not completing PROMs. These patient groups may therefore be underrepresented in outcome evaluations. This can skew the results, for example towards a more positive outcome, as sicker patients are less likely to fill out PROMs. Hence, results from PROMs with low response rates need to be interpreted with caution. This potential risk of non-response bias should be considered when using PROMs as outcome measures and in quality assurance programs.

Additionally, patients who do not complete PROMs may be deprived of the advantages in health care services associated with PROMs, such as close symptom monitoring and individually tailored treatment options. As our results indicate that patients with a non-native language background and those without supplementary health insurance have a higher risk of non-response to some PROMs, potential barriers related to language and socio-economic circumstances should be considered when implementing PROM assessments. Considering that patients with lower socio-economic status and people from immigrant backgrounds often face discrimination and disadvantages in health care [30, 31], it is particularly important to enhance the response rates for these patient groups to promote health equity.

Response rates on PROMs can be enhanced by addressing patients’ capability and motivation to participate, as suggested by van Engen et al., [37], e.g. by highlighting the purpose and benefits of PROMs. Offering PROMs in multiple languages seems especially pertinent, given the large effect of non-native language background. Furthermore, patients may be supported by accommodating specific barriers such as technical illiteracy with paper administration of PROMs [25, 37]. As clinic-level factors substantially influence PROM completion, organizational practices and best-practice approaches in PROM implementation also need to be considered [12]. However, additional support for vulnerable patient groups inevitably requires extra staff effort. This represents an ongoing challenge in high-workload environments. Technical solutions, such as AI-supported tools [15], offer promising avenues to reduce this burden while ensuring equitable patient participation.

## Conclusions

Non-response to PROMs in Swiss rehabilitation is predicted by impaired health, older age, as well as a proxy for non-native language skills, and is influenced by clinic-level factors. Barriers to PROM-completion should be reduced for patients with these characteristics to reduce the risk of non-response bias and to ensure health equity.

## Supplementary Information

Below is the link to the electronic supplementary material.

Supplementary Material 1 : Description of instruments (PROMs, CIRS), Supplementary Tables 1-4

Supplementary Material 2 : Supplementary Figure 1 - Predicted odds for non-response on the MacNew Heart, HADS, PHQ-15 and CRQ by CIRS score

Supplementary Material 3 : Supplementary Figure 2 - Predicted odds for non-response on the MacNew Heart, HADS, PHQ-15 and CRQ by length of rehabilitation

Supplementary Material 4 : Supplementary Table 5 - Descriptive characteristics by response status and instrument/PROM by year and diagnosis category (Table 1 continued)

Supplementary Material 5 : Supplementary Table 6 - Odds ratios and confidence intervals for non-response by instrument/PROM, year and diagnosis categories (Table 2 continued)

Supplementary Material 6 : Supplementary Table 7 - Descriptive characteristics by instrument/PROM of excluded patients with unclear response status

## Data Availability

Due to data protection regulations of the ANQ, the data is not publicly available. However, it is available upon request by submitting an application to the ANQ. Contact information can be found on the ANQ-Webportal (https://www.anq.ch/de/anq/kontakt).

## Footnotes

1 Since 2024, HADS has been replaced by PHQ-9 (depression module of the PHQ) and General Anxiety Disorder (GAD)-7 questionnaire.

## Abbreviations

CI: Confidence interval
CIRS: Cumulative Illness Rating Scale
CRQ: Chronic Respiratory Disease Questionnaire
HADS: Hospital Anxiety and Depression Scale
HRQOL: Health–related quality of life
ICC: Intraclass correlation coefficient
L1: First language
OR: Odds ratio
PHQ: Patient–Health–Questionnaire
PHQ-15: Somatic symptoms module of the Patient–Health–Questionnaire
PROMs: Patient–reported outcome measures
SD: Standard deviation
STROBE: Strengthening the Reporting of Observational Studies in Epidemiology
