# Supplementary material for: Predictors for non-response to PROMs across three areas of inpatient rehabilitation – a nationwide cross-sectional study in Switzerland
Source: J Patient Rep Outcomes. 2026 Aug 25;10:161. doi: 10.1186/s41687-026-01183-1 (PMC13540864; doi:10.1186/s41687-026-01183-1)
Supplement: Supplementary file 1 [file 41687_2026_1183_MOESM1_ESM.docx]

# Supplement

1. **Description of instruments**

The MacNew Heart questionnaire contains a total of 27 items on three subscales. Each item is answered on a Likert scale of 1 to 7, ranging from very high constrains to no constraints, respectively. A minimum number of items has to be completed on each subscale (7 of 14 items on the physical scale, 6 of 12 items on the emotional scale, and 7 of 13 items on the quality of social life scale) in order to calculate a final mean score across all items.

The HADS is a scale of 14 items divided into two separate subscales, an anxiety scale and a depression scale, with 7 items each. Valid entries are 0 to 3 for all items, with higher scores corresponding to higher levels of anxiety or depression. A maximum of one item per subscale may be missing and scores for the missing items are imputed from the mean of the other items. The final score for each subscale is the sum of all items on the respective scale and can each range from 0 to 21.

The PHQ-15 consists of 15 items, but only 14 items are included in the analysis for quality assurance purposes. Item 4 (menstruation pain) is excluded to allow for score comparison among menstruating and non-menstruating patients. Each item is completed with values ranging from 0 to 2 (no constraints to high constraints), which results in a final total score between 0 and 28. A maximum of three items may be missing, for which values are then imputed from the mean of all other items.

The CRQ contains 4 subscales and a total of 20 items. A minimum number of items needs to be completed on each subscale (3 out of 5 on the dyspnoe subscale, 3 of 4 items on the fatigue subscale, 4 of 7 items on the emotional functioning scale and 3 out of 4 on the mastery of disease scale). The items are answered on a Likert scale ranging from 1 to 7, with 1 indicating very high constraints and 7 indicating no constraints. Each subscore is the mean across all items on the respective scale and a total score can be calculated from the mean of the subscores.

The CIRS is a 14-item scale, rated by clinical staff, of which the final total score is a comorbidity index ranging from 0 to 56 with higher scores indicating highest comorbidity.

2. **Supplementary Tables**

Supplementary Table 1 - Diagnosis categories cardiac rehabilitation

| CATEGORY | ICD-10-CODES |
| --- | --- |
| Chronic ischaemic heart disease | I25, Q24.5 |
| Other ischaemic heart diseases | I20-I24 |
| Non-rheumatic mitral valve disorders | I34, Q23.2, Q23.3 |

Supplementary Table 1 (continued) - Diagnosis categories cardiac rehabilitation

| CATEGORY | ICD-10-CODES |
| --- | --- |
| Non-rheumatic aortic valve disorders | I35, Q23.0, Q23.1, Q23.4-Q23.9 |
| Other forms of heart disease | I30-I33, I36-52, A01.0, A18.8, A36.8, A39.5, A52.0, A54.8, B26.8, B37.6, B57.0, B57.2, B58.8, B65, D86.8, E63.9, E05.0, E85, J09, J10.8, J11.8, M05.3, M10.0, M32.1, N18, O08.8, O75.4, O90.3, O99.4, Q20-28, R00, R57.0, R94.3, S26, Z94.1, Z94.3 |
| Diseases of arteries | I70-I79, D22, G45.9, K55.0, M30-36, N28.0, Q82.5 |
| Other disorders of the circulatory system | I00-I15, I26-I28, I60-I69, I80-I99, A67.2, B74, D15.1, F01, G08, G10, G25.5, G45, G90.3, K75.1, L03, L04, N50.8, O22, O26.5, O87.8, Q82.0, Q88, R03.1, R57.9, R59, S06, S25, S35, S45, S55, S65, S75, S85, S95, T80-T82 |
| Other diseases | All other ICD-10-Codes |

Supplementary Table 2 - Diagnosis categories in psychosomatic rehabilitation

| CATEGORY | ICD-10-CODES |
| --- | --- |
| Addiction disorders | F10-F19 |
| Affective disorders without a current severe episode | F30-F39 ohne F31.4, F32.2-8 und F33.2-3 |
| Severe depressive episode | F31.4, F32.2-8, F33.2-3 |
| Difficulties in coping with life | Z73 |
| Neurotic & Stress Disorders | F40-F44, F46-F48 |
| Somatoform disorders | F45 |
| Musculoskeletal disorders & symptoms not classified elsewhere | M, R00-R50, R52-R69 |
| Headache | R51, G43, G44 |
| Other mental and somatic illnesses | All other ICD-10-Codes |

Supplementary Table 3 - Diagnosis categories Pulmonary rehabilitation

| Category | ICD-10-Codes |
| --- | --- |
| Flu & Pneumonia | J09-J18, A01.0, A02.2, A21.2, A22.1, A37, A42.0, A43.0, A48.1, A49.2, A69.8, A70, A78, B01.2, B05.2, B06.8, B25.0, B37.1, B38.0-B38.2, B39, B44.0, B44.1, B58.3, B59, B65, B77.8, G00, I00, O29.0, O74.0, O89.0, P23, P35.0 |
| COPD with FEV1 <35% | J44.00, J44.10, J44.80, J44.90 |
| COPD with FEV1 ≥35% and <50% | J44.01, J44.11, J44.81, J44.91 |
| COPD with FEV1 ≥50% or unknown | J44.02-J44.09, J44.12-J44.19, J44.82-J44.89, J44.92-J44.99 |
| Other chronic diseases of the lower respiratory tract | J40-J43, J45-J47, A15, A16, P25, Q33.4, T79.7, T81.8 |
| Other respiratory diseases | J00-J06, J30-J39, J60-J99, I26-I28 |
| Oncological diseases respiratory tract | C00-C14, C30-C39, C45.0, C45.9, C47.0, C47.3, C49.0, C49.3, C76.0, C76.1, C77.0, C78.0-CC78.4, C85.2, D00, D14.2-D14.4, D15.2, D15.7, D15.9 |
| Other diseases | All other ICD-10-Codes |

Supplementary Table 4 – Number of cases with non-response at admission, discharge or both

| Instrument | Admission | Discharge | Both |
| --- | --- | --- | --- |
| MacNew Heart | 8891 | 9395 | 6945 |
| HADS | 926 | 1542 | 632 |
| PHQ-15 | 1145 | 1912 | 755 |
| CRQ | 2707 | 3180 | 1839 |
